# Evaluation of prenatal diagnosis of isolated ventriculomegaly

**DOI:** 10.1186/1743-8454-7-S1-S11

**Published:** 2010-12-15

**Authors:** Mami Yamasaki, Masahiro Nonaka, Yohei Bamba, Chika Teramoto, Rituko Pooh

**Affiliations:** 1 Department of Neurosurgery, Osaka National Hospital, National Hospital Organization, 2-1-14 Hoenzaka, Chuo-ku, Osaka city, Osaka 540-0006, Japan; 2 Department of Nursing, Osaka National Hospital, National Hospital Organization, 2-1-14 Hoenzaka, Chuo-ku, Osaka city, Osaka 540-0006, Japan; 3 CRIFM Clinical Research Institute of Fetal Medicine PMC, 3-7, Uehommachi 7, Tennoji, Osaka 543-0001, Japan

## Background

The prenatal management of hydrocephalus with myelomeningcele(MMC) has been established in recent years. However, other types of fetal hydrocephalus so called isolated ventriculomegaly show wide heterogeneity in prognosis, as various diseases are included. For the proper counselling .it is very important to clarify this entity.

## Materials and methods

Our objective is to evaluate how to estimate the appropriate clinical outcome prenatally in isolated ventriculomegaly. Method Retrospective study, single institute (Osaka National Hospital) Materials One hundred and seventeen cases with fetal hydrocephalus were treated at Osaka National Hospital from 1992 to 2010. As 35 cases with MMC and fetal brain tumor are excluded, 82 cases were selected for this study.

## Results

Final diagnosis was as still isolated ventriculomegaly in 30 cases. Other anomaly are detected in 19cases(X-linked hydrocephalus in5, atresia of Monro in 2, corpus callosum agenesis in 3, lissencephaly in 2, other type of hereditaly hydrocephalus in 2, chromosomal anomaly in 4 and a EEC syndrome). Final diagnosis were categorized in secondary hydrocephalus in 9 cases (virus infection in 2 and fetal intracranial hemorrhage in7) and 24 cases are diagnosed as other type of malformation( holoprosencephaly in 4 ,Dandy Walker syndrome in 3 cases, Jobert syndrome in a case, arachnoid cyst in 9 cases and encephalocele in 7cases). With exclusion of 6 aborted cases and fourteen unknown cases due to too young to evaluate or lost of follow-up, final outcome are analyzed in 62 cases. Of 62cases, 11% was dead in utero or after birth, 26% showed severe retardation, 11% moderately retarded, 16% mild retarded, and 35% of case showed good outcome.

## Conclusions

For the accurate counselling to detect accompanied anomaly, fetal sonography by expert obstetricians, fetal MRI,TORCH screening test were useful for the final diagnosis. Prospective and retrospective studies to evaluate prenatal detections by using several kinds of tools and long term outcome in isolated ventriculomegaly is required.

